# Medieval injuries: Skeletal trauma as an indicator of past living conditions and hazard risk in Cambridge, England

**DOI:** 10.1002/ajpa.24225

**Published:** 2021-01-25

**Authors:** Jenna M. Dittmar, Piers D. Mitchell, Craig Cessford, Sarah A. Inskip, John E. Robb

**Affiliations:** ^1^ McDonald Institute for Archaeological Research, University of Cambridge Cambridge UK; ^2^ Department of Archaeology University of Cambridge Cambridge UK; ^3^ Cambridge Archaeological Unit, Department of Archaeology University of Cambridge Cambridge UK

**Keywords:** fracture, interpersonal violence, lifeways, sexual division of labor, violence

## Abstract

**Objective:**

To explore how medieval living conditions, occupation, and an individual's role within society impacted their risk of skeletal trauma.

**Materials:**

The skeletal remains of 314 individuals from medieval Cambridge that were buried in the parish cemetery of All Saints by the Castle (*n* = 84), the Augustinian friary (*n* = 75), and the cemetery of the Hospital of St John the Evangelist (*n* = 155) were analyzed.

**Methods:**

Macroscopic examination and plain radiographs were used to classify fracture type. The causative mechanisms and forces applied to a bone were inferred based on fracture morphology.

**Results:**

The skeletal trauma observed represents accidental injuries, likely sustained through occupational or everyday activities, and violence. The highest prevalence rate was observed on the individuals buried at All Saints by the Castle (44%, *n* = 37/84), and the lowest was seen at the Hospital of St John (27%, *n* = 42/155). Fractures were more prevalent in males (40%, *n* = 57/143) than females (26%, *n* = 25/95).

**Conclusions:**

Skeletal trauma was highest in All Saints parish burial ground, indicating that the poor, whether working urban or rurally, had the highest risk of injury. The pattern and types of fractures observed suggests that males experienced more severe traumatic events than females. However, females that were routinely involved in manual labor were also at increased risk of injury.

**Significance:**

This article enhances our understanding of how traumatic injuries differed by age, sex, and burial locations in the medieval period.

**Further research:**

Additional comparative studies in different geographical regions are needed to determine how representative these findings are.

## 1 INTRODUCTION

In recent decades, research has explored how skeletal trauma in archaeological assemblages can be used to reconstruct injury risks related to occupation, lifestyle and environment (Agnew & Justus, 2014; Judd & Redfern, 2012; Judd & Roberts, 1998, 1999; Milner et al., 2015; Redfern, 2017a; Walker, 2012). The analysis of the type and pattern of skeletal trauma can be used to determine if the injuries sustained by past peoples were the result of an accident or due to intentional violence (Grauer & Roberts, 1996; Judd & Roberts, 1999; Walker, 2001). This, in turn, provides valuable perspectives on lived experiences as different environments and lifestyles will affect the overall prevalence rate of skeletal injuries and the locations where skeletal trauma is sustained (Agnew et al., 2015). To a certain extent, it is also possible to infer the social spaces that an individual would have occupied and the activities that they would have participated in based on their burial location. By comparing individuals buried in various locations within a town, it becomes possible to identify the hazards of daily life that were experienced by individuals that occupied different spheres of medieval society. Here, we investigate the lived experiences of the inhabitants of medieval Cambridge who were buried in a normal parish cemetery, which was the normative burial site for the majority of the population, a wealthy friary and a charitable institution for the poor, by comparing evidence of skeletal trauma.

### 1.1 Medieval Cambridge

Cambridge had reached the full extent of its medieval size by the mid‐11th century, and by the 13th century was an economically thriving market town and inland river port with an agricultural hinterland (Casson et al., 2020; Cessford, fothcoming). The population by the mid‐13th century was about *c*. 2500–4000 people. The economy was largely based on agriculture and trade that was facilitated by the river. As in any medieval town, the vast majority of the individuals were laborers (see Dyer, 2009; Miller & Hatcher, 2014). These can be broadly separated into agricultural workers (such as ploughmen and shepherds), general laborers, construction workers (including carpenters, tilers, masons, and thatchers) and artisans (such as shoemakers and tailors); close to 50 trades would have been practiced in a town the size of Cambridge (Miller & Hatcher, 2014). Both men and women worked and received wages (Bardsley, 1999). A significant proportion of the town population was directly involved in agricultural activities, farming the surrounding town fields (Maitland, 1898). While most specialized occupations were dominated by men, women worked at brewing ale, washing clothes, weaving, agriculture, domestic service, and other tasks (Casson et al., 2020, table 3.1).

Medieval Cambridge was home to numerous ecclesiastical institutions, including the university, which was founded *c*. 1208–1210. The university was not a major component of the town until the late 13th to early 14th centuries. Before this, it was comprised of relatively small individual elements within the larger town and surrounding fields (Cessford, ). The presence of the university helped attract members of various religious orders, particularly friars, to Cambridge. These included the Dominicans, Franciscans, the Carmelites, the Friars of the Sack, Friars of St Mary, the Augustinians, and the Gilbertine canons regular (Cessford, forthcoming A). Over time, the growing collegiate and religious institutions in the town became increasingly important markets for many products, some of which were quite specialized. Members of religious institutions worked in varying ways. Besides praying, conducting services and pastoral duties, some were scholars while others carried out manual tasks involved in running religious houses and their estates (Andrews, 2006).

Although a small town by today's standards, Cambridge presented a varied social landscape. Gender was a social division that crosscut all socio‐economic and vocational categories. Medieval gender systems considered men and women to be fundamentally different in many ways (see Hanawalt, 1986). On the ground, this meant that different activities, forms of work and forms of interaction were considered appropriate for men and women (Hanawalt, 1986; Philips, 2003). Townspeople included a small number of prosperous families with extensive properties and servants, and a large preponderance of laboring folk. The latter may have included some pursuing specialized trades, some general laborer, and farm workers (see Dyer, 2009; Miller & Hatcher, 2014). A major adult subgroup would have been members of religious foundations such as friaries and colleges, who would have both worked at different activities and experienced a specialized lifestyle and diet governed by specified institutional rules (Ellis & Salzman, 1948). There were also the urban poor and needy, which may have included both chronically impoverished people and people driven into poverty by loss of livelihood and family support networks.

### 1.2 Activity and injury risk

During the medieval period, an individuals' occupation and the day‐to‐day activities in which they participated were influenced by their status, gender, and age (both social and chronological; see Hanawalt, 1986). These factors, along with their environment, affected an individual's injury risk. Overall, we might consider trauma risk to reflect several factors:

- Whether somebody performed manual labor, with the associated risks of accidental injury; this might imply a higher risk of trauma in an ordinary laborer rather than skilled workers, and in manual workers rather than scholars.
- Socioeconomic or institutional buffering from incidental risks of daily life (e.g. formal and informal violence); this might imply a higher risk of skeletal trauma in poorer individuals and in laypeople rather than clerics.
- The general level of aggressive or assertive interaction people were expected to experience, which might imply a higher risk of injury for males than for females.

Moreover, different groups may display not only different levels of trauma but also different patterns of injury. As men and women occupied different spheres within society and did different kinds of work, we might expect them to display different patterns of skeletal trauma. The same is true for men and women living and working in different ways, particularly ordinary people and those living in the specialized, highly regulated world of religious institutions. This is, however, an ideal model, and much of the population may also have shared the common general risks of life in a crowded medieval town. To begin to understand how levels and patterns of traumatic injuries differed by age, sex, and burial location, the human skeletal remains of the individuals that were buried in a normal parish cemetery, a wealthy friary and a charitable institution were examined. The aim of this research is to explore how the past living conditions experienced by the inhabitants of medieval Cambridge and an individual's role within society impacted their risk of injury.

## 2 MATERIALS & METHODS

### 2.1 Materials

The skeletal remains of 314 individuals from medieval Cambridge were assessed for evidence of skeletal trauma, including fractures and weapon‐related trauma. To be included in the postcranial assessment, a skeleton needed to be over 25% complete and well preserved with minimal evidence of taphonomic damage to the bone cortex. The skeletal elements in the hands and feet were not systematically recorded. The cranium was considered to be present if the frontal and both parietal bones were more than 75% complete. Over 25% of the vertebrae (six or more) and ribs (six or more) were required to be present to be included in these specific analyses. Individual ribs were assessed if they were over 50% complete, vertebrae were assessed if they were over 75% complete.

All individuals analyzed were likely to have participated in occupations and activities as adolescents or adults. The age of 12 was selected as the beginning of adolescence, as historical documentation indicates that around 12 was commonly considered to be beyond the distinct stage of childhood for much of the High/Late medieval period (1200–1500 AD) (Lewis, 2013). After 12 years of age, young people increasingly participated in adult work and activities. Skeletal remains from individuals below the age of 12 were not included in this study because 1) this research focuses upon adult activity patterns and 2) it is likely that skeletal trauma would be under‐represented in the non‐adult individuals due to the increased bone turnover during growth.

**FIGURE 1:**
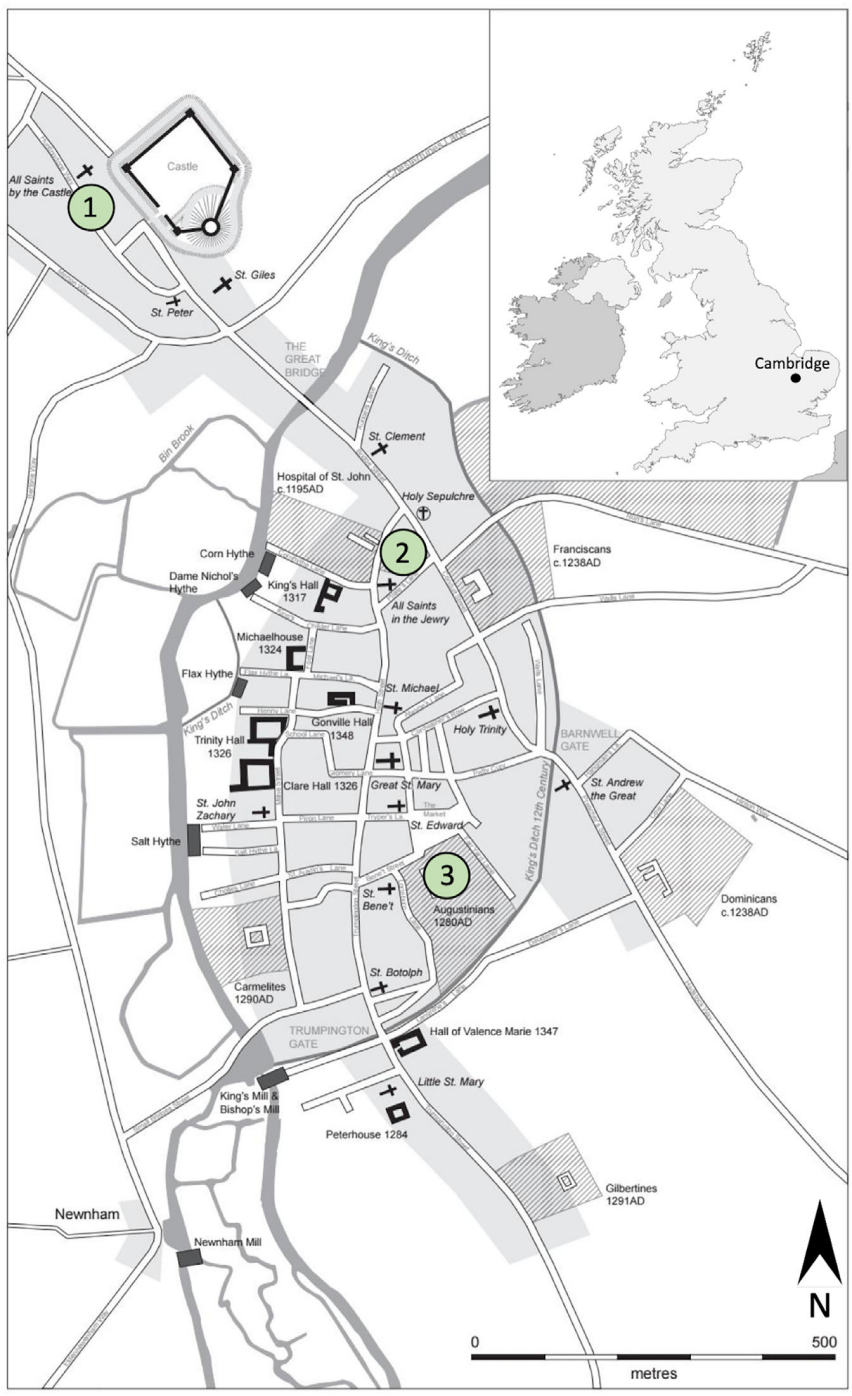
Map of Cambridge, c. 1350 indicating the location of 1) All Saints by the Castle parish burial ground, 2) the Hospital of St John the Evangelist and 3) Augustinian friary (Cambridge map by Vicki Herring; UK map inset adapted from image: Uk_outline_map.png)

By examining the data from skeletons buried in multiple locations in Cambridge, we can explore both the general living conditions for the medieval town and infer the activities and social spaces that an individual occupied. The individuals in this study are broadly representative of medieval Cambridge society. The individuals analyzed came from three sites, each representing a different social context: The parish cemetery of All Saints by the Castle, representing the majority of the townsfolk who were buried in such cemeteries, this parish was socio‐economically mixed but generally rather poorer than the town as a whole (Casson et al., 2020, p. 143–4); the Hospital of St John the Evangelist predominantly representing inmates of a charitable hospital; and the Augustinian friary representing members of the clergy and the relatively wealthy laity (Figure 1).

#### 2.1.1 The parish of All Saints by the Castle (CP73)

The parish of All Saints by the Castle, located north of the river, was likely founded *c*. 940–1100. The parish stayed in use until 1365, when it was amalgamated with a neighboring parish following population loss due to the Black Death pandemic (which affected Cambridge in 1349). The graveyard of All Saints by the Castle was identified during an archaeological evaluation in 1972 and four areas were excavated in 1973, with further skeletons uncovered in 1988 and 1994 (Cessford et al., forthcoming B). In total *c*. 215 in situ skeletons in various states of completeness have been excavated, of which 84 were analyzed here. As a parish church, All Saints represents the normal place of burial for the vast majority of ordinary people.

#### 2.1.2 The Hospital of St John the Evangelist (JDS10)

Established *c*. 1190–1200 to care for the poor and infirm of Cambridge, the Hospital of St John the Evangelist remained in use until it was dissolved to create St John's College in 1511. The burial ground was established between *c*. 1204–1214 (based on textual sources and radiocarbon dating) and reached its full extent by *c*. 1230–1250 (Cessford, forthcoming B). Textual evidence indicates that the Hospital was an ecclesiastical institution intended to provide pastoral and spiritual care for the poor and infirm (Rubin, 1987; Underwood, 2008). In 2010–2011, the burial ground associated with the Hospital was excavated by the Cambridge Archaeological Unit. The excavation unearthed 400 complete and partial in situ burials (Cessford, 2015). A total of 155 individuals from this site were examined in this study. The individuals buried in the cemetery included hospital inmates, corrodians and modest benefactors. The great majority of the individuals buried in the cemetery were likely to have been inmates (needy residents of Cambridge selected as recipients of charity including food, lodging, and clothing). They may have included people who were impoverished, chronically ill or both.

#### 2.1.3 The Augustinian friary (NRB16)

The Augustinian friary in Cambridge was established in *c*. 1279–1289 and continued in use until the Dissolution in 1538. It functioned as a *studium generale*, or national and international study house, for the Augustinian order in England (Roth, 1966). The friary acquired the right to bury members of the Augustinian order in 1290 and individuals who were not members of the friary in 1302 (Cessford, 2017). Parts of the friary were excavated in 1908–1909 and elements of *c*. 47 skeletons recovered. The dating of the skeletons recovered in 1908–1909 is uncertain, but they probably date to *c*. 1360/1420–1538 as they appear to be associated with the cloisters dated to this period. Only the skulls of these individuals are now available for analysis. In 2016–2017, the Cambridge Archaeological Unit excavated other parts of the site, and 38 further burials were uncovered (Cessford, 2017). These include 32 cemetery burials dating to *c*. 1290–1360/1420, and six additional burials from inside the chapter house, dated *c*. 1360/1420–1538. Of the individuals buried in the friary, 19 were buried clothed while wearing belt buckles (Cessford, 2017). The individuals buried with buckles are believed to have been friars; the rest are believed to be wealthier townspeople. The skeletal remains of 75 individuals were examined including 47 skulls from earlier excavations at the Augustinian friary.

### 2.2 Methods

**FIGURE 2:**
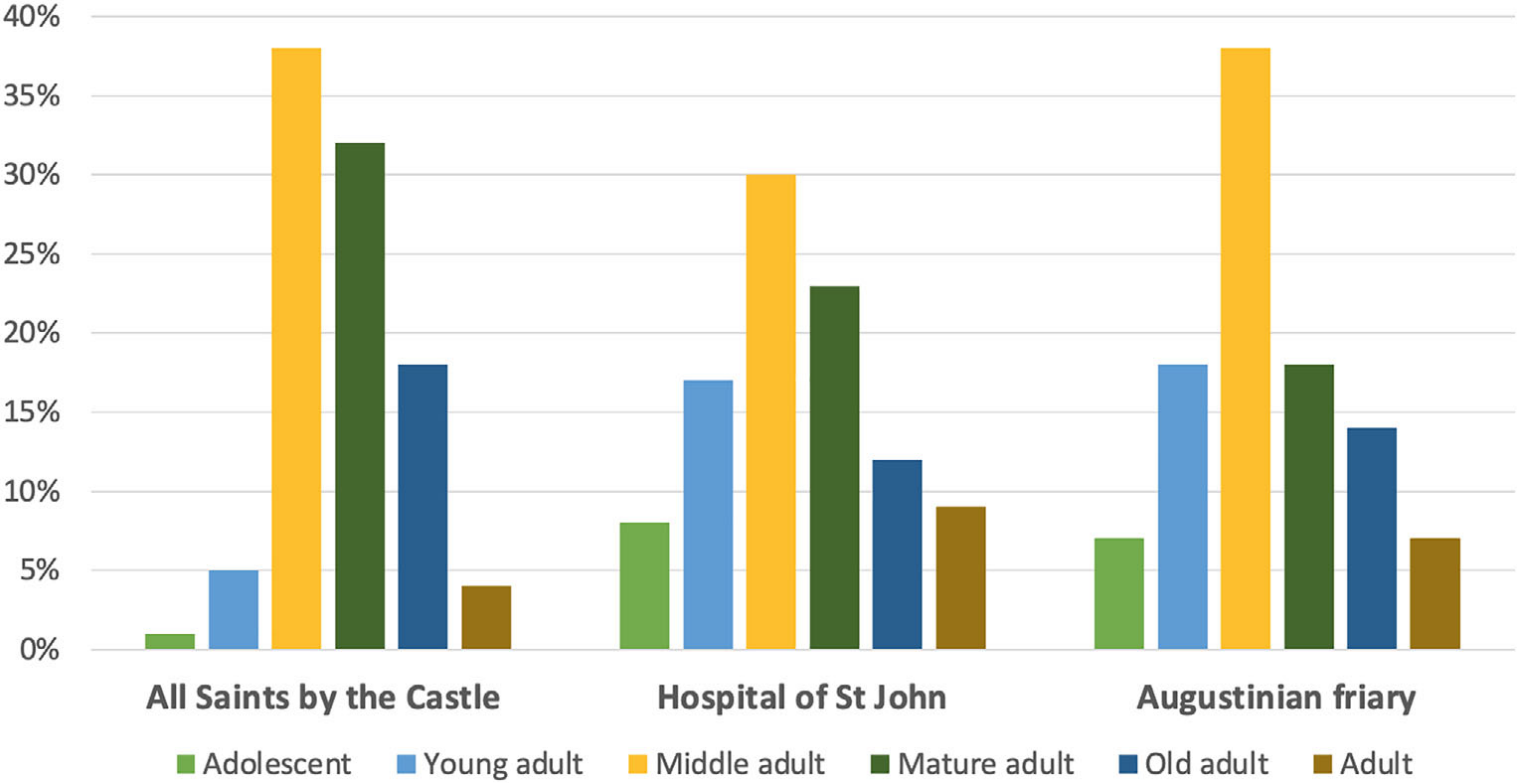
Age distribution of individuals analyzed that had postcranial elements (*n* = 267) by site

As multiple sites were examined, each skeleton was assigned a project specific number (PSN) that served as a unique identifier. The assessment of the skeletal remains followed British guidelines (Brickley & McKinley, 2004; Mitchell & Brickley, 2017). Age‐at‐death of adult individuals was estimated by assessing pubic symphyseal morphology (Brooks & Suchey, 1990), auricular surface morphology (Buckberry & Chamberlain, 2002), sternal rib ends (İşcan et al., 1984, 1985) and the sternal end of the clavicle (Falys & Prangle, 2015). The age of non‐adult individuals was determined using calcification of the permanent mandibular dentition (Moorrees et al., 1963). The biological sex of each individual over 15 years of age was estimated by examining the sexually dimorphic characteristics of the pelvis and skull (Buikstra & Ubelaker, 1994; Phenice, 1969; Schwartz, 2007) and, when available, through aDNA analyses (Inskip et al., 2019). Skeletons were divided into the following age categories: adolescent (12–17), young adult (18–25), middle adult (26–44), mature adult (45–60), and old adult (60+). If age‐at‐death could not be determined due to incompleteness or damage, individuals with complete epiphyseal fusion were classified as “adult.” Table 1 displays the sex and age‐at‐death distribution for all of the individuals included in this study. Although there are fewer adolescents and young adults from the burial ground of All Saints parish, the age distribution of the three assemblages are broadly comparable age distributions (Figure 2). As such, prevalence rates of trauma will be comparable between these sites and observed differences between these sites can be used to compare differences in risk as the result of lifestyle and occupation.

**TABLE 1:** Frequency table for biological sex and age‐at‐death categories

Age	Male	Female	Indeterminate	Unobservable	Total	Percentage of sample
*All Saints by the Castle*						
Adolescent	0	0	0	1	1	1%
Young adult	3	1	0	0	4	5%
Middle adult	16	15	1	0	32	38%
Mature adult	11	15	1	2	29	35%
Old adult	6	8	0	1	15	18%
Adult	1	1	0	1	3	4%
**Total**	**37**	**40**	**2**	**5**	**84**	
*Hospital of St John*						
Adolescent	4	2	0	7	13	8%
Young adult	12	11	0	4	27	17%
Middle adult	28	17	0	2	47	30%
Mature adult	23	12	0	1	36	23%
Old adult	11	6	0	1	18	12%
Adult	2	6	0	6	14	9%
**Total**	**80**	**54**	**0**	**21**	**155**	
*Augustinian friary*						
Adolescent	2	0	0	0	2	7% (3%)^b^
Young adult	5	0	0	0	5	18% (7%)^b^
Middle adult	10	0	0	0	10	38% (13%)^b^
Mature adult	5	0	0	0	5	18% (7%)^b^
Old adult	3 (5)	1 (1)	0	0	4 (6)	14% (13%)^b^
Adult	1 (37)	0 (3)	0	1 (1)	2 (41)	7% (57%)^b^
**Total**	**26 (42)**	**1 (4)**	**0**	**1 (1)**	**28 (75)** ^a^	

a Indicates the total number of individuals from the friary including the 47 additional skulls from the 1908‐09 excavation.

b Indicates percentage of sample including the skulls from the 1908 excavation.

If identified, elements with skeletal trauma were described according to Lovell (1997) and selected elements were further analyzed using plain radiography (X‐ray). X‐rays were taken by Reveal X‐ray Imaging Solutions using a portable DR‐Go X‐ray machine. The frequency of each fracture was calculated by specific location on each skeletal element. When appropriate, chi‐squared or Fisher's exact tests were used to determine statistical significance. Significance level was set at *p* < 0.05.

If evidence of healing was present, a fracture was classed as antemortem. If no evidence of healing was present, the fracture was classed as perimortem. Perimortem fractures were distinguished from postmortem damage based on the coloration of the bone at the fracture, the location of the injury and the fracture morphology (Galloway & Wedel, 2014; Sauer, 1998). The causative mechanisms and the forces applied to a bone can be inferred by the type of break that has occurred (see Galloway et al., 2014; Redfern & Roberts, 2019). Observed fractures were classed as: transverse, oblique, spiral, comminuted, impacted, greenstick, avulsion or compression. Transverse fractures are complete breaks that run perpendicular to the shaft that are caused by a combination of tension and compression stresses. Fractures of this type can be associated with high‐energy mechanisms or direct force (Rogers, 1992). Oblique fractures extend diagonally across the bone (with an angle ≥30° perpendicular to the long axis) and are caused by compressive and shearing stresses (Galloway et al., 2014; Meinberg et al., 2018). Spiral fractures are complete fractures caused by torsion, which result in the bone twisting apart (Galloway et al., 2014). Oblique and spiral fractures are indicative of higher energy mechanisms; often related to falls or jumps from a height (Johner et al., 2000). Comminuted fractures are those where the bone breaks into multiple pieces and can result from a range of high impact mechanisms. An impacted fracture is a complete fracture where the broken ends are driven into each other so that the fracture line is indistinct. Such fractures are usually the results from compression (Galloway et al., 2014). Greenstick fractures are incomplete fractures that affect only one side of a bone as the result of bending from the opposite side. Greenstick fractures are only seen in children's long bones (Galloway et al., 2014). Avulsion fractures occur when a tendon or ligament pulls off a piece of bone. Compression fractures occur as the result of a sudden impaction (Redfern & Roberts, 2019). As fractures may be the result of more than one type of stress, it was not always possible to classify a fracture into one of these categories, especially if substantial healing had occurred. In these cases, the type was classed as unobservable. Based on this classification, traumatic injuries were also classed as resulting from direct trauma (when a fracture occurs at the point of impact), and indirect trauma (where the fracture is located not at the point of impact; Lovell, 1997).

The timing (ante‐ or perimortem), the patterning, and the possible mechanism of the observed skeletal trauma was assessed to attribute the probable cause to one of the following categories: (1) accident‐ and occupation‐related trauma and (2) trauma that is likely to have been the result of interpersonal violence. Sharp‐force trauma and any type of trauma to the skull is more likely to be indicative of aggression‐related trauma rather than accidental or occupational injuries (Walker, 2001). There are several postcranial injuries that are associated with interpersonal violence, such as parry fractures, isolated fractures to the shaft of the ulna, and fractures of the ribs and hands and feet (Humbyrd et al., 2012). Although the presence of any of these injuries in isolation are not necessarily indicative of interpersonal violence (Redfern, 2017b; Walker, 1989).

Skeletal trauma accumulates over an individuals' life, as individuals who have lived longer will have an increased likelihood of accumulating antemortem injuries. As such, skeletal assemblages with a greater number of older adult individuals present are likely to show higher rates of healed trauma than those with a higher percentage of younger individuals, even if their actual risk of being injured in any one year is the same or lower. Age‐related loss of bone mass also increases the likelihood of fractures in older individuals (Cummings & Melton, 2002). Individuals with osteoporosis, a systemic skeletal disease that is characterized by severely reduced bone mineral density and deteriorated bone microarchitecture, have a significantly increased risk of fractures (Cummings & Melton, 2002; Lorentzon, 2019). Classic osteoporotic fractures occur in the vertebrae, proximal femur (hip), and the distal forearm, but clinical research has shown that almost all types of fractures are increased in patients with low bone density (Cummings & Melton, 2002; Kanis et al., 2013). However, due to the limitations of diagnosing osteopenia and osteoporosis in archeological human skeletal remains, individuals with these conditions were not identified within this work.

## 3 RESULTS

Of the individuals with postcranial elements, 32% (*n* = 86/267) displayed one or more fractures. No evidence of sharp‐force trauma was observed. Fractures were most commonly found on the individuals buried in the All Saints parish cemetery (44%, *n* = 37/84), followed by those buried in the Augustinian friary burial ground (32%, *n* = 9/28) and in the burial ground of the Hospital of St John (27%, *n* = 42/155; Table 2). Of note, 15% (*n* = 40/267) of individuals had two or more fractures. Individuals with multiple fractures were most common at All Saints parish cemetery (19%, *n* = 16/84), followed by those buried at the Augustinian friary (14%, n = 4/28) and the Hospital (13%, *n* = 20/155). Nearly all fractures occurred antemortem; only one individual from the Augustinian friary displayed clear perimortem trauma.

**TABLE 2:** Frequency and true prevalence rate of fractures by skeletal elements and site

	All Saints by the Castle			Hospital of St John			Augustinian friary			Town total	Total prev.
	Total #	Fractured	Prev.	Total #	Fractured	Prev.	Total #	Fractured	Prev.		
*Axial*											
Cranium	47	4	**9%**	65	4	**6%**	67	2	**3%**	10/179	**6%**
Right scapula	67	1	**2%**	93	2	**2%**	26	0	**0%**	3/186	**2%**
Left scapula	69	1	**1%**	105	2	**2%**	26	0	**0%**	3/200	**2%**
Right clavicle	65	1	**2%**	90	0	**0%**	24	0	**0%**	1/179	**1%**
Left clavicle	63	2	**3%**	93	0	**0%**	24	0	**0%**	2/180	**1%**
Ribs^a^	74	15	**20%**	118	20	**17%**	24	4	**17%**	40/216	**19%**
Vertebra (body)^a^	71	8	**11%**	126	9	**7%**	24	2	**8%**	19/221	**9%**
Vertebrae (spinous process)^a^	71	0	**0%**	126	6	**5%**	24	1	**4%**	7/221	**3%**
*Upper limb*											
**Right humerus**											
Proximal epiphysis	58	0	**0%**	72	0	**0%**	24	0	**0%**	0/154	**0%**
Proximal shaft	62	0	**0%**	86	0	**0%**	24	0	**0%**	0/172	**0%**
Midshaft	64	0	**0%**	88	0	**0%**	24	0	**0%**	0/176	**0%**
Distal shaft	63	0	**0%**	88	0	**0%**	24	0	**0%**	0/175	**0%**
Distal epiphysis	60	0	**0%**	87	1	**1%**	24	0	**0%**	1/171	**1%**
**Left humerus**											
Proximal epiphysis	54	0	**0%**	86	0	**0%**	27	0	**0%**	0/167	**0%**
Proximal shaft	59	0	**0%**	96	0	**0%**	27	0	**0%**	0/182	**0%**
Midshaft	58	0	**0%**	102	0	**0%**	27	0	**0%**	0/187	**0%**
Distal shaft	60	0	**0%**	101	1	**1%**	27	0	**0%**	1/188	**1%**
Distal epiphysis	60	0	**0%**	99	2	**2%**	27	0	**0%**	2/186	**1%**
**Right radius**											
Proximal epiphysis	61	0	**0%**	92	0	**0%**	25	0	**0%**	0/178	**0%**
Proximal shaft	63	0	**0%**	100	0	**0%**	25	0	**0%**	0/188	**0%**
Midshaft	60	0	**0%**	103	0	**0%**	25	0	**0%**	0/188	**0%**
Distal shaft	59	1	**2%**	101	1	**1%**	24	0	**0%**	2/184	**1%**
Distal epiphysis	58	0	**0%**	98	0	**0%**	23	0	**0%**	0/179	**0%**
**Left radius**											
Proximal epiphysis	65	0	**0%**	100	0	**0%**	26	0	**0%**	0/191	**0%**
Proximal shaft	68	0	**0%**	109	0	**0%**	25	0	**0%**	0/202	**0%**
Midshaft	69	0	**0%**	104	0	**0%**	25	0	**0%**	0/198	**0%**
Distal shaft	64	1	**2%**	109	1	**1%**	25	0	**0%**	2/198	**1%**
Distal epiphysis	60	1	**2%**	105	0	**0%**	24	0	**0%**	1/189	**1%**
**Right ulna**											
Proximal epiphysis	68	1	**1%**	92	1	**1%**	23	0	**0%**	2/183	**1%**
Proximal shaft	70	0	**0%**	102	0	**0%**	25	0	**0%**	0/197	**0%**
Midshaft	67	0	**0%**	102	0	**0%**	25	0	**0%**	0/194	**0%**
Distal shaft	63	0	**0%**	98	2	**1%**	25	0	**0%**	1/186	**1%**
Distal epiphysis	58	1	**2%**	82	1	**1%**	20	0	**0%**	2/160	**1%**
**Left ulna**											
Proximal epiphysis	65	0	**0%**	105	0	**0%**	27	0	**0%**	0/197	**0%**
Proximal shaft	67	0	**0%**	110	0	**0%**	27	0	**0%**	0/204	**0%**
Midshaft	66	20	**0%**	106	2	**2%**	27	0	**0%**	2/199	**1%**
Distal shaft	65	2	**3%**	104	0	**30%**	27	1	**4%**	3/196	**2%**
Distal epiphysis	62	0	**0%**	92	0	**0%**	22	0	**0%**	0/176	**0%**
*Lower limb*											
**Right femur**											
Proximal epiphysis	72	1	**1%**	103	1	**1%**	26	0	**0%**	2/201	**1%**
Proximal shaft	70	0	**0%**	105	0	**0%**	26	0	**0%**	0/201	**0%**
Midshaft	70	0	**0%**	101	0	**0%**	26	1	**4%**	1/197	**1%**
Distal shaft	70	0	**0%**	101	0	**0%**	27	0	**0%**	0/198	**0%**
Distal epiphysis	67	0	**0%**	95	1	**1%**	25	0	**0%**	1/187	**1%**
**Left femur**											
Proximal epiphysis	71	0	**0%**	114	0	**0%**	25	0	**0%**	0/210	**0%**
Proximal shaft	72	0	**0%**	120	0	**0%**	27	0	**0%**	0/219	**0%**
Midshaft	70	0	**0%**	117	0	**0%**	27	1	**4%**	1/214	**1%**
Distal shaft	70	0	**0%**	118	0	**0%**	27	0	**0%**	0/215	**0%**
Distal epiphysis	66	0	**0%**	116	0	**0%**	27	0	**0%**	0/209	**0%**
Right patella	49	0	**0%**	51	1	**2%**	22	0	**0%**	1/122	**1%**
Left patella	46	0	**0%**	56	1	**2%**	24	0	**0%**	1/126	**1%**
**Right tibia**											
Proximal epiphysis	57	0	**0%**	86	0	**0%**	24	0	**0%**	0/167	**0%**
Proximal shaft	58	0	**0%**	86	0	**0%**	24	0	**0%**	0/168	**0%**
Midshaft	55	0	**0%**	85	0	**0%**	25	0	**0%**	0/165	**0%**
Distal shaft	53	0	**0%**	82	0	**0%**	25	0	**0%**	0/160	**0%**
Distal epiphysis	51	0	**0%**	77	1	**1%**	25	0	**0%**	1/153	**1%**
**Left tibia**											
Proximal epiphysis	58	1	**2%**	95	0	**0%**	24	0	**0%**	1/177	**1%**
Proximal shaft	58	0	**0%**	94	0	**0%**	25	0	**0%**	0/177	**0%**
Midshaft	59	0	**0%**	91	0	**0%**	25	0	**0%**	0/175	**0%**
Distal shaft	56	0	**1%**	92	1	**1%**	25	0	**0%**	1/173	**1%**
Distal epiphysis	52	0	**0%**	85	0	**0%**	25	0	**0%**	0/162	**0%**
**Right fibula**											
Proximal epiphysis	42	1	**1%**	60	0	**0%**	19	0	**0%**	1/121	**1%**
Proximal shaft	53	0	**0%**	88	0	**0%**	25	0	**0%**	0/166	**0%**
Midshaft	56	0	**0%**	87	0	**0%**	25	0	**0%**	0/168	**0%**
Distal shaft	57	1	**4%**	87	0	**0%**	25	0	**0%**	1/169	**1%**
Distal epiphysis	51	2	**4%**	74	0	**0%**	22	0	**0%**	2/147	**1%**
**Left fibula**											
Proximal epiphysis	41	0	**0%**	70	0	**0%**	24	0	**0%**	0/135	**0%**
Proximal shaft	54	0	**0%**	96	0	**0%**	25	0	**0%**	0/175	**0%**
Midshaft	55	0	**0%**	94	1	**1%**	25	0	**0%**	1/174	**1%**
Distal shaft	54	0	**0%**	91	0	**0%**	25	0	**0%**	0/170	**0%**
Distal epiphysis	49	0	**0%**	83	0	**0%**	22	0	**0%**	0/154	**0%**

a Indicates the number of individuals with fractures, not the total number of fractures.

Fractures were more commonly found on males (40%, *n* = 57/143) than females (26%, *n* = 25/95), but this difference was not statistically significant when analyzed using a Chi‐square test (*p* = 0.08). Fractures were most prevalent in old adults, with 59% (*n* = 22/37) of individuals having one or more fractures. A higher percentage of old adult males had fractures (70%, *n* = 14/20) than old adult females (53%, *n* = 8/15). Similar trends were identified in mature and middle adults (see Table 3).

**TABLE 3:** Age and sex distribution of skeletal trauma in medieval Cambridge

Age	Male	Female	Indeterminate	Unobservable	Total
*All Saints by the Castle*					
Adolescent	–	–	–	0/1	0/1
Young adult	0/3	0/1	–	–	0/4
Middle adult	9/16	4/15	1/1	–	14/32
Mature adult	6/11	7/15	1/1	0/2	14/29
Old adult	4/6	5/8	–	0/1	9/15
Adult	0/1	0/1	–	0/1	0/3
**Total**	**19/37** (51%)	**16/40** (40%)	**2/2** (100%)	**0/5** (0%)	**37/84 (**44%)
*Hospital of St John*					
Adolescent	0/4	0/2	–	1/7	1/13
Young adult	2/12	1/11	–	0/4	3/27
Middle adult	11/28	3/17	–	0/2	14/47
Mature adult	10/23	2/12	–	0/1	12/36
Old adult	7/11	2/6	–	0/1	9/18
Adult	1/2	0/6	–	1/6	2/14
**Total**	**31/80** (39%)	**8/54** (15%)	–	**2/21** (8%)	**41/155** (26%)
*Augustinian friary* ^a^					
Adolescent	0/2	–	–	–	0/2
Young adult	0/5	–	–	–	0/5
Middle adult	1/10	–	–	–	1/10
Mature adult	3/5	–	–	–	3/5
Old adult	3/3	1/1	–	–	4/4
Adult	0/1	–	–	0/1	0/2
**Total**	**7/26** (27%)	**1/1** (100%)	–	**0/1** (0%)	**8/28** (29%)

a The skeletal remains from the 1908‐09 excavation are not included in this table, as they have no associated post crania.

### 3.1 Cranial and maxillofacial trauma

A total of 179 crania were assessed, including the isolated skulls from the early excavations at the Augustinian friary (*n* = 47). 9% (*n* = 4/47) of the individuals from All Saints parish had antemortem blunt‐force trauma compared with 6% (*n* = 4/65) from the cemetery at the Hospital and 3% (*n* = 2/67) from the Augustinian friary (Table 4). All fractures to the cranial vault in these assemblages were small, well‐healed depression fractures in the external table with no further involvement other than a reduction of diplöic space. No evidence of weapon‐related trauma was observed. When analyzed collectively, males (6%, *n* = 7/118) and females (5%, *n* = 3/58) had similar prevalence rates of trauma to the cranial vault.

**TABLE 4:** Demographics of individuals with trauma to the cranium by site

	Male			Female			Unknown sex (incl. Unobservable and indeterminate)			Total	Total prev.
	All Saints by the Castle (*n* = 18)	Hospital of St John (*n* = 39)	Augustinian friary (*n* = 61)	All Saints by the Castle (*n* = 28)	Hospital of St John (*n* = 25)	Augustinian friary (*n* = 5)	All Saints by the Castle (*n* = 1)	Hospital of St John (*n* = 1)	Augustinian friary (*n* = 1)		
Adolescent	–	0/3	0/2	–	0/2	–	0/1	0/1	–	**0/9**	**0%**
Young adult	1/3	1/9	0/5	0/4	0/10	–	–	–	–	**2/31**	**7%**
Middle adult	1/3	0/7	0/7	0/4	0/5	–	–	–	–	**1/26**	**4%**
Mature adult	0/5	2/12	1/4	1/11	1/5	–	–	–	–	**5/37**	**14%**
Old adult	0/6	0/7	0/6	1/7	0/2	0/2	–	–	–	**1/30**	**3%**
Adult, age unknown	0/1	0/1	1/37	0/2	0/1	0/3	–	–	0/1	**1/46**	**2%**
**Total**	**2/18**	**3/39**	**2/61**	**2/28**	**1/25**	**0/5**	**0/1**	**0/1**	**0/1**	**10/179**	**6%**
**Total Prev.**	**11%**	**8%**	**3%**	**7%**	**4%**	**0%**	**0%**	**0%**	**0%**	**6%**	

Maxillofacial trauma was observed on two individuals. One adult male buried in the cemetery of the Hospital of St John and one adult female from All Saints parish burial ground (PSN 705) had antemortem fractures to the body of the mandible. Direct trauma sustained through assaults and falls are the most common cause of mandibular fractures (Morris et al., 2015). In modern times, mandibular fractures occur much more frequently in men than in women.

### 3.2 Postcranial trauma

#### 3.2.1 Shoulder girdle

Nine fractures to skeletal elements in the shoulder girdle were observed in six individuals (Table 2). Six of these fractures were located on the scapula and three fractures were located on the clavicle. Two individuals (PSN 737, 738) both old adult males from All Saints parish burial ground, had fractured both their scapula and clavicle. Fractures to the shoulder girdle were more commonly found in men (*n* = 4) than in women (*n* = 1); it was not possible to estimate the biological sex of one individual.

The individual with the isolated clavicle fracture may have sustained this injury from a fall onto the shoulder, from a direct blow, or from falling onto an outstretched hand (Humbyrd et al., 2012; Lovell, 1997). Scapular fractures are uncommon, even in modern times and are usually caused by direct trauma, or from falls onto the shoulder (Humbyrd et al., 2012). Direct trauma is the most likely cause for four of the individuals with antemortem fractures to the acromion process (*n* = 3) and to the blade of the scapula (*n* = 2; Lovell, 1997). In the clinical literature, fractures to the blade of the scapula are most frequently observed in motor vehicle collisions with high‐energy impacts, excessive muscle contractions from electrocution, or sporting activities (Imatani, 1975; Nordqvist & Petersson, 1995). As such, scapular fractures are usually associated with multiple injuries including severe injuries to the chest and skull (McGahan et al., 1980). This is illustrated by the extensive injuries sustained by an old adult male buried in the All Saints parish (PSN 737) who had antemortem fractures to the blade of the left scapula, the left clavicle and 11 ribs on the left side, including the first rib. Most of the left side ribs had more than one fracture. Given the patterning of these injuries, and the context from which the remains come, it is likely that these injuries were the result of blunt force trauma to the thoracic cavity, possibly due to a fall from a height, or from being crushed or pinned by livestock.

#### 3.2.2 Rib fractures

Ribs were the most commonly fractured element at all three sites (see Table 5). A total of 19% (*n* = 40/216) of individuals that had ribs present had at least one fractured rib; All Saints parish burial ground rib fractures (20%, *n* = 15/74), Hospital of St John (17%, *n* = 20/118), and the Augustinian friary (17%, *n* = 4/24). Biological sex was estimated for 201 individuals with ribs. More males (21%, *n* = 25/119) had rib fractures than females (15%, *n* = 12/82). This was not a statistically significant difference (*p* = 0.46). Of the individuals buried in All Saints parish cemetery, rib fractures were present on 24% (*n* = 8/34) of males and 16% (*n* = 6/37) of females. Similarly, 23% (*n* = 14/62) of males from the Hospital had fractured ribs compared to only 11% (*n* = 5/44) of females. When the laterality of rib fractures was explored, no significant difference was found in the prevalence rate of fractures between the right and left side (*p* = 0.11).

**TABLE 5:** Prevalence rate of individuals with rib fractures by biological sex in burial locations in medieval Cambridge

	Male			Female			Indeterminate			Unobservable			Total
	All Saints by the Castle(*n* = 34)	Hospital of St John (*n* = 62)	Augustinian friary (*n* = 23)	All Saints by the Castle (*n* = 37)	Hospital of St John (*n* = 44)	Augustinian friary (*n* = 1)	All Saints by the Castle (*n* = 2)	Hospital of St John (*n* = 0)	Augustinian friary (*n* = 0)	All Saints by the Castle (*n* = 1)	Hospital of St John (*n* = 10)	Augustinian friary (*n* = 0)	
Adolescent	–	0/4	0/3	–	0/2	–	–	–	–	–	1/5	–	1/14
Young adult	2/9	1/15	0/5	1/6	1/17	–	1/1	–	–	–	0/4	–	6/60
Middle adult	0/9	4/16	0/8	1/10	1/7	–	–	–	–	–	–	–	6/47
Mature adult	5/9	5/19	1/4	3/12	3/11	–	0/1	–	–	–	–	–	17/56
Old adult	1/6	4/7	2/3	2/9	0/5	1/1	–	–	–	–	0/1	–	10/32
Adult, age unknown	0/1	0/1	–	–	0/2	–	–	–	–	0/1	0/2	–	0/7
**Total**	**8/34**	**14/62**	**3/23**	**6/37**	**5/44**	**1/1**	**1/2**	–	–	**0/1**	**1/12**	–	**40/216**

**FIGURE 3:**
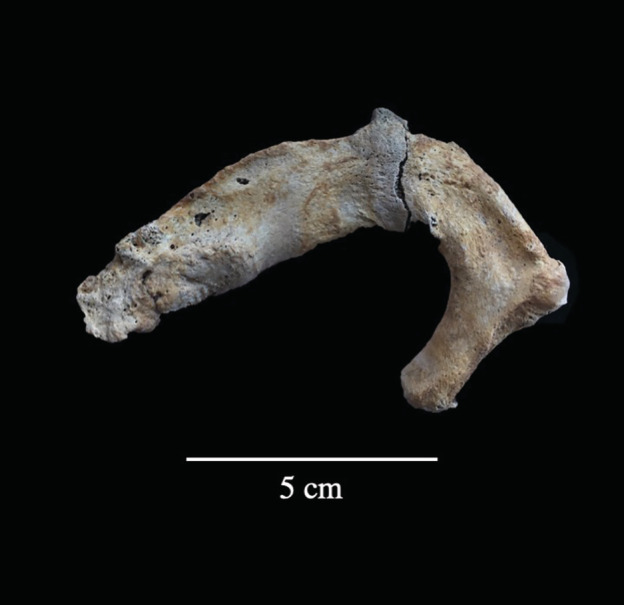
Superior view of an antemortem fracture on the first rib of an adult male individual (PSN 57) from the Hospital of St John, Cambridge. Photograph by Jenna Dittmar

Individuals with multiple rib fractures, or with at least one fractured rib and a fracture located elsewhere, may have experienced blunt‐force trauma to the thoracic region as the result of a fall, an assault or a work‐related injury (see Sirmali et al., 2003; Ziegler & Agarwal, 1994). Within this subgroup, 45% (*n* = 18/40) individuals had multiple fractured ribs, and 48% (*n* = 19/40) of individuals had at least one fractured rib and at least one fracture located elsewhere on the body. Two adult male individuals had fractures to the first rib (PSN 57 and PSN 737) in addition to other post‐cranial elements (Figure 3). Individuals that had multiple fractured elements, including ribs were primarily mature and old adults (*n* = 15/19), with males and females equally affected. In males, rib fractures were most commonly present in individuals with fractures to the appendicular skeleton or with fractures associated with direct trauma (such as scapular fractures). This pattern was not observed in females, where rib fractures were most commonly present in individuals with compression fractures in the vertebra.

#### 3.2.3 Fractures to the long bones

**FIGURE 4:**
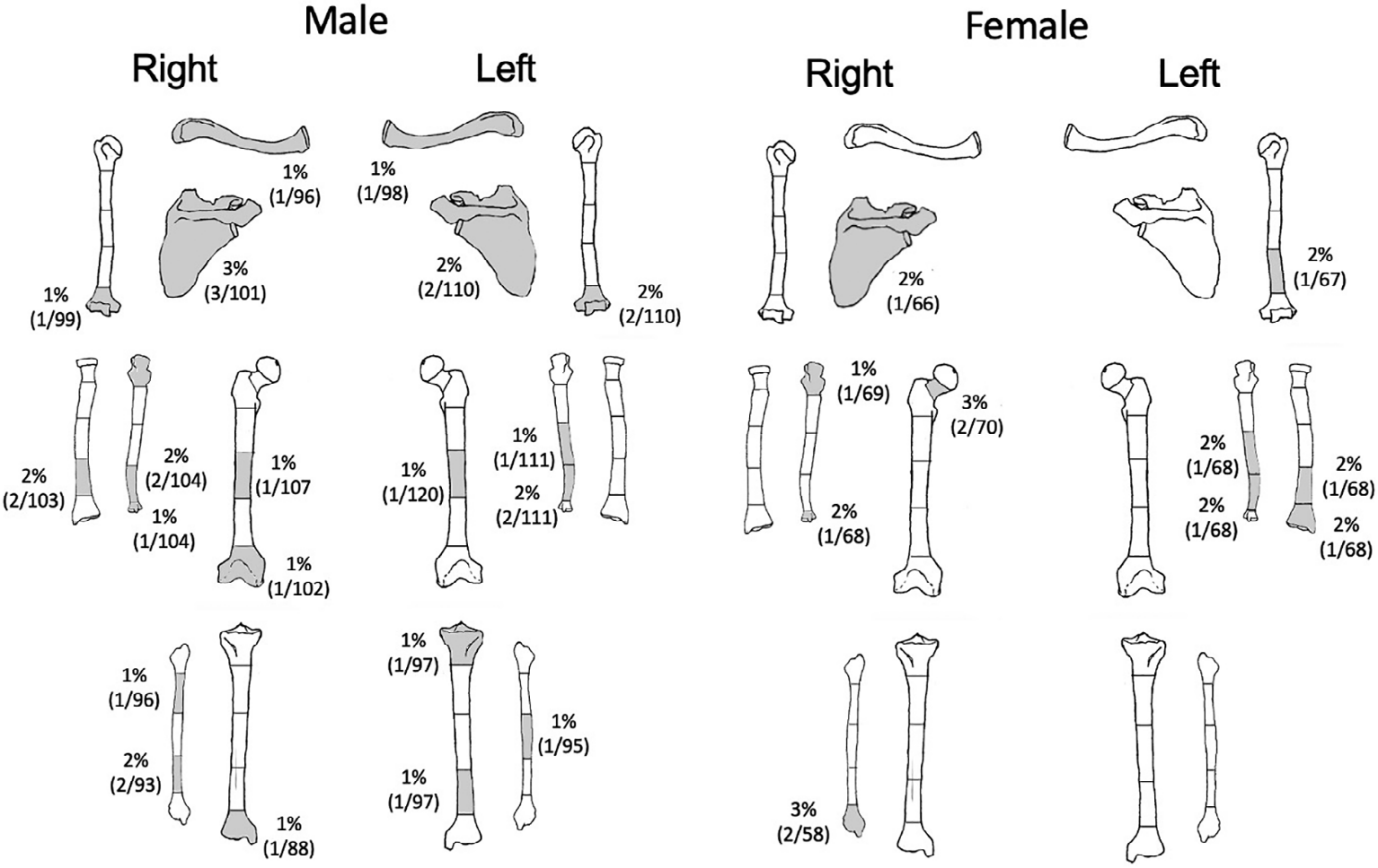
Visual representation of the distribution and true prevalence rate of fractures in females (*n* = 96) and males (*n* = 143)

A total of 33 long bone fractures were identified on 11% (*n* = 29/267) of individuals (Tables 3 and 6). A higher percentage of males had fractures than females (Figure 4). The majority of these fractures occurred antemortem, but one individual from the Augustinian friary had perimortem bilateral comminuted fractures to the femoral shafts (first reported in Neil, 2017). Comminuted fractures are usually caused by high‐energy injuries and are commonly associated with more extensive injuries to the soft tissue (Sölveborn, 2014).

**TABLE 6:** Fractures in long bones (by type) observed. Fracture type was recorded as unobservable in cases of extensive healing

PSN	Site	Sex	Age	Element	Side	Location of fracture	Fracture type	Associated injuries	Possible mechanism	Fracture timing
49	Hospital of St John	M	Mature adult	Femur	Right	Distal condyle	Hairline	Hairline fracture to left patella	Fall onto knees	Antemortem
84	Hospital of St John	M	Young adult	Ulna	Right	Distal 1/3 of shaft	Unobs.		Direct trauma	Antemortem
92	Hospital of St John	M	Old adult	Ulna	Right	Distal 1/3 of shaft	Oblique	Fractures to the acromion process of scapula, shafts of right second metacarpal and fourth left metacarpal, left ribs (3)	Fall	Antemortem
93	Hospital of St John	M	Old adult	Tibia	Left	Distal 1/3 of shaft	Spiral	Rib fracture	Twisting with left foot planted	Antemortem
				Fibula	Left	Midshaft	Spiral			Antemortem
115	Hospital of St John	M	Mature	Tibia	Right	Distal articular surface	Hairline		Stress fracture	Antemortem
156	Hospital of St John	M	Young Adult	Ulna	Left	Midshaft	Unobs.	Right fifth metatarsal	Direct trauma	Antemortem
158	Hospital of St John	M	Young Adult	Ulna	Right	Coronoid process	Transverse tip fracture		Fall onto elbow	Antemortem
160	Hospital of St John	M	Middle adult	Humerus	Right	Medial epicondyle	Avulsion		Throwing, valgus force	Antemortem
191	Hospital of St John	M	Middle adult	Humerus	Left	Medial epicondyle	Avulsion		Throwing, valgus force	Antemortem
230	Hospital of St John	F	Old adult	Ulna	Left	Midshaft	Oblique		Fall onto outstretched hand	Antemortem
244	Hospital of St John	M	Middle adult	Radius	Right	Distal 1/3 of shaft	Unobs.		Fall onto outstretched hand	Antemortem
335	Hospital of St John	F	Mature adult	Femur	Right	Neck	Unobs.	Fracture to right patella	Secondary to osteoporosis, possible fall	Antemortem
				Ulna	Right	Styloid process	Transverse		Fall onto outstretched hand	Antemortem
346	Hospital of St John	F	Mature adult	Radius	Left	Distal 1/3 of shaft	Unobs.	Fracture on shaft of right third metacarpal	Fall onto outstretched hand	Antemortem
353	Hospital of St John	M	Old adult	Humerus	Left	Supracondylar fracture	Oblique		Fall on to outstretched hand during childhood	Antemortem
357	Hospital of St John	F	Mature	Humerus	Left	Distal 1/3 of shaft	Oblique		Possible fall on to outstretched hand	Antemortem
510	Augustinian friary	M	Adult	Ulna	Left	Distal 1/3 of shaft	Transverse	BFT to Cranium, antemortem fracture to base of fifth metacarpal	Direct trauma	Antemortem
531	Augustinian friary	M	Middle adult	Femur	Left	Midshaft	Comminuted	Perimortem fracture to T1 and C6	High energy trauma	Perimortem
				Femur	Right	Midshaft	Comminuted			Perimortem
698	All Saints by the Castle	F	Middle adult	Radius	Left	Distal articular facet	Unobs		Fall onto outstretched hand	Antemortem
713	All Saints by the Castle	F	Mature adult	Fibula	Right	Distal articular surface	Oblique		Rotation of the foot in relation to the leg	Antemortem
718	All Saints by the Castle	F	Mature adult	Ulna	Left	Distal 1/3 of shaft	Unobs.		Direct trauma	Antemortem
719	All Saints by the Castle	F	Mature adult	Fibula	Right	Distal lateral malleolus	Transverse	Avulsion fracture on calcaneus	Rotation of the foot in relation to the leg	Antemortem
723	All Saints by the Castle	M	Old adult	Tibia	Left	Plateau	Compression		Possible, jump or fall from height in which knee forced into varus during childhood	Antemortem
729	All Saints by the Castle	F	Old adult	Femur	Right	Neck	Unobs.		Secondary to osteoporosis	Antemortem
730	All Saints by the Castle	M	Middle adult	Radius	Right	Distal 1/3 of shaft	Transverse		Fall onto outstretched hand	Antemortem
				Ulna	Right	Styloid process	Transverse			Antemortem
738	All Saints by the Castle	M	Old adult	Fibula	Right	Proximal 1/3 of shaft	Oblique	Fractured right clavicle (midshaft), acromion process of right scapula, compression fracture in vertebra (L3)	Direct trauma	Antemortem
743	All Saints by the Castle	I	Middle adult	Radius	left	Distal 1/3 of shaft	Oblique	Fractured left clavicle	Fall onto outstretched hand	Antemortem
770	All Saints by the Castle	M	Young adult	Fibula	Right	Distal 1/3 of shaft	Oblique		Rotation of the foot in relation to the leg	Antemortem
772	All Saints by the Castle	M	Young adult	Ulna	Left	Distal 1/3 of shaft	Transverse	Right fifth metacarpal, left rib	Direct trauma or fall	Antemortem
778	All Saints by the Castle	F	Mature adult	Ulna	Right	Coronoid process	Transverse tip fracture		Fall onto elbow	Antemortem

**FIGURE 5:**
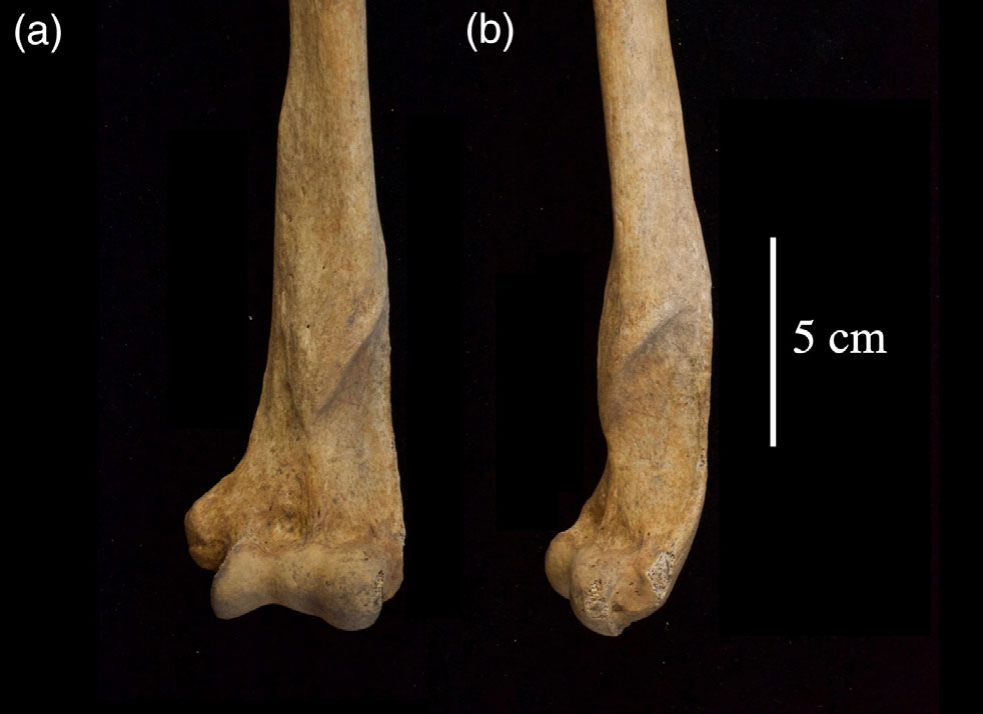
Anterior view of an antemortem fracture of distal left humerus from a mature adult female (PSN 357), the Hospital of St John, Cambridge. Photograph by Jenna Dittmar

Fractures to the upper limb were the most common (*n* = 20), many of which can be attributed to falls. Colles' fractures to the distal radius, with dorsal angulation and dorsal displacement, were identified on three individuals (M = 2, F = 1). These fractures result from a fall forward onto an outstretched arm. Transverse tip fractures of the coronoid process of the ulnae were observed on two individuals. This type of fracture is the result of shearing forces that typically occur as the distal humerus is driven against the coronoid, usually in a fall. Often, this type of fracture is associated with elbow dislocation but can occur in isolation (Regan & Morrey, 1989). One individual had fractures to the right ulna and radius, which likely occur as the result of a fall on an outstretched arm. Four individuals had fractures to the humerus; two had fractures to the distal third of the shaft that likely occurred as the result of a fall (Figure 5), two had avulsion fractures to the medial epicondyle. This injury can occur in adolescence as the result of throwing but can also occur if valgus force is exerted with the elbow resulting in the dislocation of the elbow (Adams, 1965; Kilfoyle, 1965; Knüsel, 2011). Isolated ulnar shaft fractures were observed on seven individuals. Such injuries can result from a rotational force to the wrist (Galloway et al., 2014) or they can be the result of a direct blow to the forearm as a victim attempts to protect themselves against an overhead blow (Humbyrd et al., 2012; Sölveborn, 2014). Isolated transverse ulnar fractures that are located on the distal half of the shaft and which have minimal displacement are called parry, or nightstick fractures (Judd, 2008). This type of fracture can be the result of a direct blow to the forearm (Judd, 2008). At least three, but possibly as many as five individuals, had parry fractures.

**FIGURE 6:**
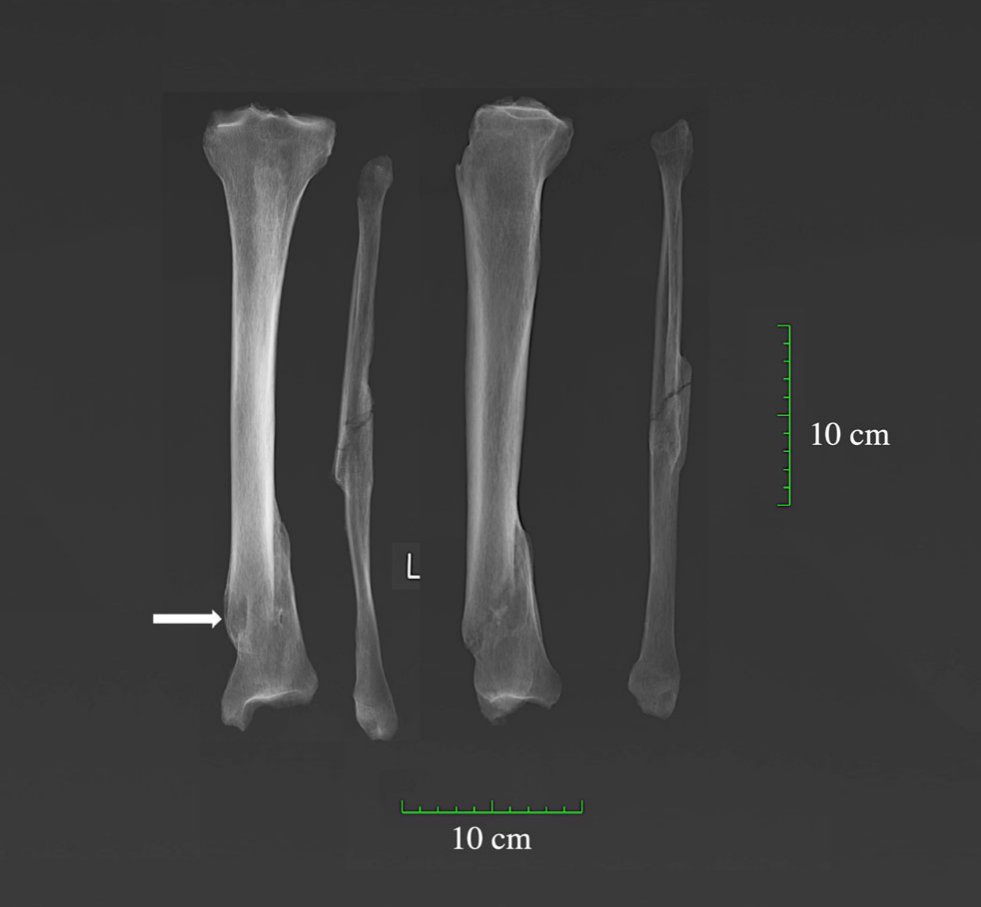
X‐ray of antemortem spiral fracture of the tibia and fibula complicated by osteomyelitis (arrow indicates the cloaca) on an adult male (PSN 93) from the Hospital of St John, Cambridge. X‐rays taken by Reveal Imaging, image created by Jenna Dittmar

Fractures to the lower limb, probably caused by a variety of mechanisms, were observed in 11 individuals. One mature male (PSN 49) had hairline fractures to the medial facet of left patella and to the medial condyle of right femur, likely resulting from a fall onto the knees. Two female individuals (PSN 335, 729) had intracapsular fractures to the neck of the femur. This type of hip fracture is a common osteoporosis‐related fracture in the elderly that can occur from falls (Sölveborn, 2014). One old adult male (PSN 723) had a well‐healed tibial plateau fracture that resulted in a substantial limb length discrepancy. Fractures that involve the tibial plateau occur when a force drives the lower end of the femur into the tibial plateau, such as jump or a fall from a height (Sölveborn, 2014). Another male individual (PSN 93) had spiral fractures on the tibia and fibula; this commonly occurs while the body is in rotational motion, but one foot is planted on the ground (Figure 6). Transverse fractures to the fibulae, most often caused by direct trauma, were observed in two individuals. One individual (PSN 738) has a proximally located isolated fibular fracture that was most likely caused by direct trauma to the fibula (Humbyrd et al., 2012). Ankle injuries, often due to rotation of the foot in relation to the leg (Humbyrd et al., 2012), were found on three individuals (PSN 713, 719, 770) from All Saints parish burial ground.

### 3.3 Age differences in trauma

Substantially more adult individuals who died over the age of 60 showed evidence for antemortem skeletal trauma. This is partly explained by biological factors such as age‐related bone loss which contributes to the higher prevalence rate of antemortem fractures in older adult individuals as weaker bones are more prone to fracture during a fall (Johnell & Kanis, 2005; Riggs et al., 2006). Several individuals had fractures that most commonly occur in older adults with osteoporosis such as compression fractures in the vertebrae (*n* = 18) and fractures to the femoral neck (intracapsular fractures; *n* = 2) (see Kanis et al., 2013). Both of the individuals with hip fractures were old adult females. In modern populations, fractures to this region have been shown to increase exponentially with age and are often associated with underlying pathology such as osteoporosis (Johnell & Kanis, 2005; Riggs et al., 2006). As this data suggests, older individuals in these samples had different levels of trauma, not only because they accumulated skeletal trauma throughout their life, but also because they experienced new life risks with older age.

## 4 DISCUSSION

### 4.1 Accidental trauma and intentional violence

The location and patterning of the skeletal trauma present on the vast majority of the skeletons appear consistent with accidental and occupational‐related trauma. Both males and females had traumatic injuries that reflect hazards associated with the individual's living environment, such as falls (see Table 6). The types of fractures observed on male individuals suggests that some experienced severe traumatic events (including fractures to first and second ribs, scapulae, and clavicle). Males were also more likely to have fractures that are commonly caused by direct trauma. During the medieval period, direct trauma could have been be caused by another person (interpersonal violence), or could have been inflicted by livestock, tools (such as mallets, spades, and hoes), equipment (carts, plows), and building materials such as wooden beams or stones. Females primarily had trauma consistent with indirect trauma from falls, twisted ankles, and those that typically result from decreased bone mineral density (e.g. compression fractures in the spine, hip fractures).

Antemortem fractures in locations that are commonly associated with interpersonal violence, including the cranial vault (*n* = 10), mandible (*n* = 2), the blade of the scapula (*n* = 3), parry fractures (*n* = 5), and to multiple ribs from the same individual (*n* = 19), were identified. The patterning of fractures was examined in each of these cases, as this can aid in the differentiation between accidental and violent trauma. In most of these cases, there is not sufficient skeletal evidence to infer interpersonal violence. However, a few individuals including both of the individuals that exhibit fractures to the body of the mandible, were likely involved in interpersonal violence as damage to the cranial vault and facial bones is often an indicator, as is multiple trauma. One of these individuals was an old adult female from the All Saints parish burial ground (PSN 705) who had numerous antemortem fractures; the fracture morphology and the location of the trauma observed provides additional support for an interpretation of interpersonal violence. Besides the mandibular fracture, antemortem fractures were also located on multiple ribs, on the base of the right first metatarsal, and multiple vertebra. All of these injuries were well healed, and it was not possible to determine if these injuries occurred during a single event or from multiple episodes. Although a fall or severe accident cannot be excluded as possible causes (Allan & Daly, 1990), the most common cause of injuries to the head, neck and face, including fractures to the facial bones in women is assault (Brink et al., 1998; Zachariades & Papavassiliou, 1990). Given the patterning of the observed injuries, it is possible that these injuries were sustained as the result of domestic, or intimate partner violence.

Numerous historical studies have investigated violence, including domestic or intimate partner violence during the medieval period (see Butler, 2007; Hanawalt, 1986; Jones, 2006). Some, including Hanawalt (1986) have argued against the commonly held belief that spousal abuse was common (see Hanawalt, 1986). Research on coroners' rolls showed that spousal homicide, albeit an extreme form of spousal abuse, was very uncommon (Butler, 2001: 63; Hanawalt, 1986) with only 0.7% of felonies involving an individual committing a crime against a member of their family (Hanawalt, 1986). This is further supported by data derived from rolls from the manorial courts which list very few cases (2%) that detail disputes between members of the same family (Hanawalt, 1986: 208; Hanawalt, 1976: p. 309). Much more frequently, these sources show husbands and wives working together towards common goals. The skeletal evidence from this study provides support for this argument as very little evidence of domestic violence was observed. However, it is likely that the vast majority of intimate partner violence will have gone undocumented as only the most severe cases of abuse would have appeared before the courts (Butler, 2007). Typically, domestic disputes were viewed as a community matter and were addressed by members of the family or neighbors (Hanawalt, 1986). As such, reconstructing an accurate picture of abuse and maltreatment during the medieval period remains challenging.

Research on data derived from coroners' rolls from the 14th and 15th centuries suggests that physical violence was commonplace and people, particularly men, were quick to defend themselves violently (Hanawalt, 1979: p. 273; Jones, 2006: p. 63). It has been said that homicide was so common that in London and Oxford an individual was more likely to be murdered than die from an accident (Hanawalt, 1979: p. 99). Although the records of law courts and coroners' rolls suggest that violence was rife during the medieval period, this study found no evidence of sharp‐force trauma. Although interpretation of this finding is limited by the lack of soft tissue, it may be that bladed weapons such as daggers and knives, were not commonly used to commit violent acts in Cambridge. A finding that is contradictory to previous research on the medieval period that showed that weapons that cut or pierced were used in 73% of murders, with knife wounds causing 42% of fatalities (Hanawalt, 1976: p. 310).

As warfare was a common feature of the medieval landscape, the lack of antemortem sharp‐force trauma within this study is particularly interesting. Previous research has estimated that 2% (*n* = 133/6283) of individuals from the medieval period have skeletal trauma that has been inflicted by a weapon (Roberts & Cox, 2003). Numerous wars took place throughout the medieval period and it is highly unlikely that no one from Cambridge participated in these events. The lack of weapon trauma may suggest that if an individual survived a battle, they either did so unscathed or they did not return to Cambridge. It is also possible that individuals with weapon‐trauma were buried elsewhere within the town. Further research on this topic will need to await the excavation of additional medieval burial grounds within Cambridge.

### 4.2 Life in medieval Cambridge

Evidence of skeletal trauma was highest in the All Saints parish burial ground, indicating that ordinary laboring folk, particularly the poorer members of society, whether working in urban or rural contexts, had the highest risk of injury.

During this time, the household was the center of economic production and all members of a household would work together to sustain themselves (Howell, 1986). In addition to a residence, the household also acted as the primary location for market production (goods or services to sell). In rural areas like Cambridgeshire, many people would have been part‐time specialists, combining craft production with small‐scale farming (Dyer, 2009). Similar to small‐scale farming during modern times, farming was not a distinct occupation, but a lifestyle that requires a variety of activities to be performed by all of the occupants. As such, all occupants were at risk of injury including children (see Cogbill et al., 1991; Vane et al., 1993; Wilk, 1993). Factors predictive of increased injury risk in agricultural settings include working with large animals, and the amount of time spent engaged in farm work (see Carruth et al., 1972; Cogbill et al., 1991). The division of labor within households may partly explain the variation in the pattern and types of fractures observed between the males and females. The greater number of fractures observed in males may be due to the greater total hours engaged in agricultural work and the performance of more hazardous jobs compared to women.

### 4.3 Sexual division of labor and fracture risk

More males in Cambridge showed evidence for skeletal trauma than females; a finding that is consistently reported throughout the literature on the medieval period (Burrell et al., 2018; Judd & Roberts, 1999; Mays et al., 2007; Walker, 2012). In a large‐scale survey consisting of 58 English medieval burial grounds, significantly more males (19%, *n* = 995) than females (14%, *n* = 459) had fractures (Grauer & Miller, 2017). At the rural parish cemetery of Wharram Percy, 54% of the males compared to 43% of the females had skeletal fractures (Mays et al., 2007). A similar trend was identified by Judd and Roberts (1999) in the rural burial ground at Raunds Furnells, where 22% of the males but only 17% of the females displayed fractures. This trend was not limited to the rural areas. Based on the assessment of individuals from 10 cemeteries in London, 24% (*n* = 580/2404) of males compared to 18% of females (*n* = 277/1566) had evidence of skeletal trauma (Grauer & Miller, 2017). Similarly, the assessment of the individuals buried in four cemeteries within York revealed that the prevalence rate of skeletal trauma for men was almost twice that of women; 16% of males (*n* = 580/2404) versus 9% of females, respectively (Grauer & Miller, 2017). Historically, it has been argued that there was a division of labor that involved women working within the household and men working outside of it. In reality, this was often not the case. Both men and women contributed to the household economy, yet they did not necessarily perform the same tasks (Howell, 1986). This is supported by research on rural medieval British sites by Judd and Roberts (1999), which found that the location and types of fractures suggested segregation of activities performed by men and women.

The data from Cambridge suggests that not all women were equally likely to sustain skeletal trauma. More females buried in the parish (40%, *n* = 16/40) had evidence of skeletal trauma than did the females buried at the Hospital (15%, *n* = 8/42). This is a statistically significant difference (*p* = 0.0056), which may be explained by differences in the activities commonly undertaken by women within disparate spheres of society. Typically, women participated in a wide variety of tasks as they were expected to marry, run a household and care for children (Mate, 1999). Women living in rurban areas (areas with a mixture of urban and rural activities), such as the those buried in the All Saints parish cemetery, would have been responsible for household duties including childcare, meal preparation and laundry (Mate, 1999), but would have also been involved in the production of the materials needed within the household, involved in the harvest, caring for animals, gardening and other “outdoor” activities (Howell, 1986; Mate, 1999). The women living in the Hospital, which was established to care for the “poor and infirm” under the Augustinian rule, may have not participated in these everyday activities (Cessford, 2015; Rubin, 1987: p. 157). Relatively little is known about how the women at the Hospital would have participated in everyday chores. However, as many of the individuals in the Hospital had skeletal evidence of chronic illnesses such as tuberculosis, it is likely that they would have been unable to perform activities that involved manual labor. Even if they only spent the final period of their lives as inmates of the Hospital, they may have been unable to work strenuously for some time before their admission. The difference between sites thus probably does not reflect socioeconomic differences, but differences between women who were actively working members of society and experienced the life risks this involved, and women who were poor, needy, and often ill with long‐term chronic diseases.

### 4.4 The contemplative life of the cleric?

Of the 19 individuals believed to be friars based on the presence of belt buckles in their graves, six had evidence of trauma. The members of the friary were primarily engaged in spiritual activities and studying, as well as being involved in the public life of the church in their localities, organizing and encouraging processions and other festivities (Andrews, 2006: pp. 69–172; Lawrence, 2015). But as part of monastic life, the friars also performed daily activities that contributed to the running and upkeep of the friary. Such tasks and activities varied greatly between religious orders but were often highly ritualized and included manual labor (Olson, 2013). The performance of the tasks required to sustain the monastery were typically conducted in the afternoons, as the mornings were generally reserved for attending High Mass and other religious activities (Lawrence, 2015). As well as for getting practical jobs done, manual labor was perceived as necessary for health and exercise, and it was insisted upon in most monastic orders (Mays, 2009).

**FIGURE 7:**
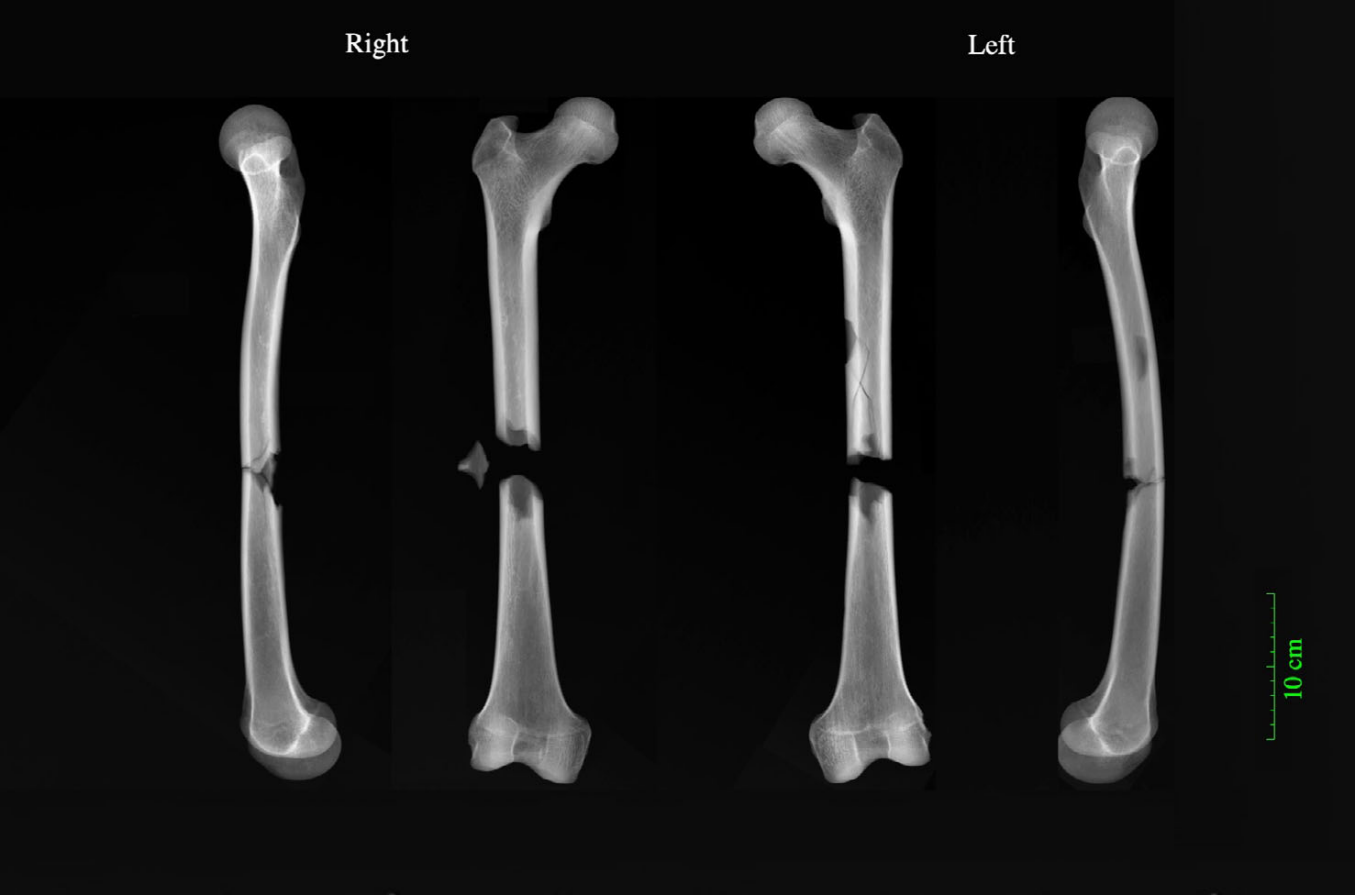
X‐rays of perimortem butterfly fractures to both femora of an adult male (PSN 531) buried in the Augustinian friary, Cambridge. X‐rays taken by Reveal Imaging, image created by Jenna Dittmar

Three of the friars had trauma that was likely accidental and may have resulted from accidents that occurred while undertaking such manual tasks, including antemortem fractures to the ribs and in the vertebral column (i.e., fractured spinous process). However, the type and pattern of injuries observed on two friars suggest that the members of this religious order did not always have sheltered, protected lives. One friar (PSN 531) had perimortem bilateral comminuted femoral fractures that indicate that he was involved in a severe, likely lethal, accident (Figure 7; first reported in Neil, 2017: p. 87). Butterfly fractures are commonly seen in the lower extremities when the thigh or calf receives a lateral blow during weight bearing for instance, among pedestrians injured by automobiles (Gozna et al., 1982). The fractures observed here are the result of direct blunt‐force trauma and could have been the result of this individual being struck, possibly by a cart. Another friar (PSN 510) had ante‐mortem fractures that may have been the result of interpersonal violence including an antemortem fracture to the distal left ulna and blunt‐force trauma to the cranium. Although it is possible that the observed fractures were accidental, it is not possible to rule out interpersonal violence. The advanced stage of healing makes it impossible to determine when these injuries occurred, and it is possible that this individual may have entered into order after he sustained these injuries.

### 4.5 Pathology & fracture risk

Fracture risk within these assemblages will undoubtedly have been affected by underlying pathological conditions (see Ives & Brickley, 2014; Judd & Roberts, 1998), early‐life health (Parsons et al., 1997), nutrition (Weisz & Albury, 2013), and reproductive behaviors (Mays, 2006). An unspecified number of individuals within this study will have experienced periods of famine that will have likely affected their health status, including the Great Famine (1315–1317). As malnutrition and starvation during childhood and early adulthood can have lasting effects on bone demineralization which predispose an individual to osteoporosis later in life (Weisz & Albury, 2013), it is likely that the individuals in this study may have an increased risk of fractures. Further research is required to investigate such issues (see Ives & Brickley, 2014).

## 5 CONCLUSION

Through the examination of skeletal remains buried in multiple locations in Cambridge, we have explored the general living conditions for the medieval period and inferred the activities and social spaces that an individual would have occupied based on their burial location. The fracture prevalence rates observed at these three sites suggests that the various inhabitants of medieval Cambridge all experienced risk of injury in their everyday lives. The skeletal trauma observed likely represents injuries sustained through accidental and occupational‐related activities as well as interpersonal violence.

Evidence of skeletal trauma was highest in All Saints parish burial ground, indicating that the poor, whether working in an urban or rural setting, had the highest risk of injury. However, those residing in the friary were not impervious to physical injury, interpersonal violence or death as the result of a severe accident. The site with the lowest fracture prevalence was the Hospital of St John. This is likely partly due to the numerous ways that people came to reside in the Hospital. At any given time, the population of the Hospital may have included chronically ill and frail individuals that were not engaging in risky activities, as well as those who came to reside there only for a short period prior to their death.

The higher prevalence of fractures in males is consistent with previous research that has been conducted on other skeletal assemblages from other medieval burial grounds in England (see Judd & Roberts, 1999; Mays et al., 2007; Walker, 2012), indicating that medieval men were at increased risk of injuries than were medieval women. The pattern and types of fractures observed in the males suggests that they experienced more severe traumatic events than did females. Males were also more likely to have fractures that are caused by direct trauma. However, the prevalence rate of skeletal trauma was significantly higher in the females buried at All Saints parish church than in the Hospital of St John. This suggests that poor women that were routinely involved in manual labor were also at an increased risk of injury.

## AUTHOR CONTRIBUTIONS

**Jenna Dittmar:** Conceptualization; data curation; formal analysis; investigation; methodology; writing‐original draft; writing‐review and editing. **Piers Mitchell:** Funding acquisition; methodology; supervision; writing‐original draft; writing‐review and editing. **Craig Cessford:** Data curation; funding acquisition; project administration; writing‐original draft; writing‐review and editing. **Sarah Inskip:** Data curation; formal analysis; investigation; methodology; project administration; writing‐review and editing. **John Robb:** Data curation; funding acquisition; methodology; project administration; supervision; writing‐review and editing.

## CONFLICT OF INTEREST

The authors declare no conflict of interest.

## Data Availability

The data that support the findings of this study are available from the corresponding author [JD] upon reasonable request.
